# Draft Genome Sequence of *Marinobacter* sp. Strain P4B1, an Electrogenic Perchlorate-Reducing Strain Isolated from a Long-Term Mixed Enrichment Culture of Marine Bacteria

**DOI:** 10.1128/genomeA.01617-15

**Published:** 2016-01-21

**Authors:** Victor G. Stepanov, Yeyuan Xiao, April J. Lopez, Deborah J. Roberts, George E. Fox

**Affiliations:** a Department of Biology and Biochemistry, University of Houston, Houston, Texas, USA; b School of Engineering, University of British Columbia, Kelowna, BC, Canada

## Abstract

The perchlorate-reducing strain *Marinobacter* sp. strain P4B1 was isolated from a long-term perchlorate-degrading enrichment culture seeded with marine sediment. The draft genome of *Marinobacter* sp. P4B1 is comprised of the bacterial chromosome (3.60 Mbp, G+C 58.51%, 3,269 predicted genes) and its associated plasmid pMARS01 (0.14 Mbp, G+C 52.95%, 165 predicted genes).

## GENOME ANNOUNCEMENT

The gamma proteobacterial genus *Marinobacter* (*Alteromonadaceae*) encompasses several dozens of moderately halophilic bacterial species, primarily of marine origin. Members of this genus exhibit substantial metabolic versatility, which makes them attractive for biotechnological exploitation. The salt-tolerant *Marinobacter* sp. strain P4B1 was isolated from a perchlorate-degrading mixed microbial culture (1). This culture was derived from a marine sediment sample and used in decontamination pilot studies on perchlorate- and nitrate-laden wastewater (2). The strain uses perchlorate, nitrate, and oxygen as electron acceptors, with preference for perchlorate over nitrate under anaerobic conditions. As a component of a microbial fuel cell, the strain couples anodic acetate oxidation with oxygen reduction on the cathode and thus produces electric current. Sequencing and analysis of the *Marinobacter* sp. P4B1 genome was undertaken in order to elucidate the genetic underpinnings of the strain’s perchlorate-reducing and electrogenic properties and to obtain further insights into its metabolic capabilities.

Here, we report the draft genome sequence of *Marinobacter* sp. P4B1. Genomic DNA was converted to two shotgun libraries with insert sizes of 250 to 300 bp and 350 to 750 bp, respectively, using the Illumina TruSeq DNA PCR-free sample preparation kit LT (Illumina, San Diego, CA) according to the manufacturer’s instructions. Sequencing was performed on Illumina HiSeq 2500 instruments at MD Anderson Center for Cancer Epigenetics Solexa Sequencing Core (Houston, TX) and the University of Arizona Genetics Core (Tucson, AZ). In total, 26,198,647 pairs of 75-mer reads and 11,702,836 pairs of 100-mer reads were generated. The reads were processed with Sickle 1.33, Trimmomatic 0.32 (3), and DeconSeq 0.4.3 (4) to trim terminal low-quality nucleotides, remove low overall quality reads, and filter out irrelevant sequences. Cleaned reads were assembled into contigs using the ABySS 1.5.2 *de novo* assembler (5). The assembly produced 15 contigs with a total length of 3,740,877 bp at 1,348× coverage, and an average G+C content of 58.30%. The contigs vary in size from 2,053 to 1,236,531 bp, with mean length of 249,391 bp and *N*_50_ of 378,721 bp. Gene search and annotation were carried out using the NCBI Prokaryotic Genomes Automatic Annotation Pipeline, followed by manual editing. During annotation, it was found that one of the contigs corresponds to a 144,935-bp plasmid designated pMARS01. The annotation revealed 3,207 protein-encoding genes, 4 rRNA operons, 46 tRNA genes, 1 transfer-messenger RNA (tmRNA), and 3 other noncoding RNA genes in the bacterial chromosome. The pMARS01 plasmid harbors another 165 protein-encoding genes.

Among the predicted genes, those involved in perchlorate reduction are of primary interest. They were found grouped in a *pcrABCDE*-*cld* chromosomal gene cluster. This cluster consists of genes coding for a molybdopterin-dependent oxydoreductase subunit (*pcrA*), an iron-sulfur cluster-containing subunit (*pcrB*), a tetraheme cytochrome C554 electron-transfer protein (*pcrC*), a putative chaperon assembling the perchlorate reductase catalytic complex (*pcrD*), a cytochrome *c*-type NapC-like protein (*pcrE*), and a chlorite dismutase (*cld*).

### Nucleotide sequence accession numbers.

The *Marinobacter* sp. P4B1 genomic sequence has been deposited in GenBank under the accession number LLXM00000000. The version described in this paper is the first version, LLXM01000000.
